# As infâncias da literatura: diálogos com a obra de José Martí, Horacio Quiroga, Mário de Andrade e Clarice Lispector no contexto latino-americano

**DOI:** 10.1590/0102-311XPT086024

**Published:** 2024-09-20

**Authors:** Marcelo de Abreu Maciel

**Affiliations:** 1 Universidade Federal Fluminense, Niterói, Brasil.

*A Cruzada das Crianças: Intelectuais, Cultura e Política na América Latina*, da professora Alejandra J. Josiowicz ^1^, é um trabalho que permite muitas possibilidades de leitura. Escrito originalmente como uma tese de doutorado, defendida na Universidade de Princeton (Estados Unidos) em 2013 e publicada no Brasil em 2023, o trabalho apresenta uma pesquisa extensa e muito bem conduzida sobre como a infância é discutida nas obras de José Martí (1853-1895), Horacio Quiroga (1878-1937), Mário de Andrade (1893-1945) e Clarice Lispector (1920-1977).

O livro apresenta uma introdução que posiciona o tema da infância entrelaçado às suas circunstâncias sociopolíticas e culturais, deixando claro que o assunto será tratado em sua historicidade e não de forma neutra ou universalizante. Na sequência, o trabalho segue uma trilha que nos conduz ao universo literário dos autores pesquisados, permitindo uma apresentação que costura um pouco de suas vidas pessoais, os caminhos e estilos literários, os posicionamentos editoriais diante do próprio objeto livro e a atmosfera social, histórica e cultural que contorna todo o cenário latino-americano, no qual essas obras foram estabelecidas. Importante destacar, ainda, que o livro contém notas de rodapé que indicam uma farta bibliografia para aprofundamento sobre os escritores escolhidos pela autora, a partir de outros trabalhos e pesquisas. Muitas vezes são notas que apresentam um contraponto, uma outra opinião em relação ao que se passa no texto principal, produzindo uma leitura ampliada e diversificada.

A pesquisa não busca nas obras de Martí, Quiroga, Andrade e Lispector uma infância na perspectiva do desenvolvimento. Por mais que esses aspectos possam comparecer, o livro mostra, primordialmente, como o tema da infância, ao ser tomado como uma rede discursiva de análise no panorama literário desses autores, nos permite pensar o lugar da criança nas práticas cotidianas e suas transformações sociais, culturais, políticas e familiares entre os séculos XIX e XX. Como dirá a autora: “*a infância tal como aparece nas reflexões de uma seleção de intelectuais de países latino-americanos, como construção cultural e histórica e figura do imaginário intelectual*” (p. 22).

Sendo assim, ao caminhar pela literatura de Martí, Quiroga, Andrade e Lispector, Josiowicz nos mostra não só as mudanças na construção de uma estética da infância, mas as inquietações de um cenário latino-americano que afetará o lugar da criança na sociedade e suas instituições.

Nesse sentido, o livro não perde de vista que, a partir do século XVIII, a infância foi tomada por uma série de discursos que a transformaram em objeto de interesse de pesquisas, mas, sobretudo, de controle de sua trajetória, do seu corpo e de sua presença nos diversos seguimentos da vida pública, principalmente na perspectiva médica, jurídica e pedagógica, como podemos ler nos trabalhos de Patto ^2^, Gondra ^3^, Foucault ^4^ e no próprio trabalho de Josiowicz. Assim sendo, “*pensar a infância, problematizando-a como uma invenção, permite perceber sua construção histórica como categoria das ciências do homem e a forma como ela é engendrada no contexto social moderno*” ^5^ (p. 7).

São nas transformações desse contexto que percebemos um tensionamento entre as esferas públicas e privadas em relação ao lugar da criança. Há um circuito de filiação parental, intrafamiliar, mas há também o registro de como a criança vai habitando seu lugar cívico na sociedade. É uma relação em que essas esferas se tocam, deixando claro que não existe um espaço estanque entre o que se passa no ordenamento da casa familiar e o que está posto no campo social.

Assim, a autora nos mostra que na literatura de Martí, Quiroga, Andrade e Lispector, a criança e o seu mundo não são um mero objeto de eleição e análise literária. Por meio da infância e suas circunstâncias, esses autores mostram ou denunciam aspectos das engrenagens sociais do governo da infância ^5^ na sociedade. Por exemplo, quem é a criança, para José Martí, na Cuba ainda como colônia espanhola e em seu projeto de libertação? Ou então, como perceber a infância na obra de Clarice Lispector, atravessada por saberes oriundos do campo psicanalítico, já presente no continente americano? O que é a infância para Quiroga e a crise na modernização da sociedade do início do século XX? Como ver a criança dentro do panorama estético e cultural pensado por Mário de Andrade?

Por meio da escrita desses autores, seja em seus trabalhos literários ou jornalísticos, dimensionamos, então, as adversidades da criança no campo social. Podemos perceber, no decorrer de suas obras, como a infância é pensada, representada e escrita em diversos textos que circulam no plano de uma sociedade, mostrando claramente o entrelaçamento entre literatura, cultura e política. Sendo assim, narrativas sobre a infância e a juventude não devem ser abstraídas de como e onde estão sendo produzidas. Vejamos: por que em determinado momento, por exemplo, na história do Brasil, chamávamos crianças pobres, periféricas e abandonadas de “menores” ^6^? Que estética utilizamos para representar pai, mãe, família, filho e filha em livros didáticos em determinados momentos da história educacional do país? Enfim, a maneira como enunciamos e escrevemos sobre crianças e jovens em textos literários, passando pela mídia até chegarmos aos textos oficiais das políticas públicas, pode ser uma maneira de lermos como as crianças estão sendo vistas, tratadas e representadas em um dado momento da história, no qual o tema da infância insurge.

Ao final do livro encontramos um capítulo inédito para essa edição. O capítulo cinco - *Saúde e Doença das Crianças Latino-Americanas: Entre Políticas Públicas e as Práticas Culturais* - é escrito para sua edição em português, dentro de um cenário sanitário arrasado subjetivamente e politicamente pela pandemia de COVID-19. Escrever esse capítulo demonstra uma sintonia entre o que foi narrado anteriormente no livro e os efeitos da pandemia na América Latina, principalmente quando falamos de crianças e famílias mais pobres e periféricas que tiveram suas vidas comprometidas do ponto de vista alimentar, escolar, sanitário e econômico.

Nesse capítulo, a autora encaminha uma apresentação e discussão sobre como foi se constituindo o campo das políticas públicas para a saúde infantil na América Latina, mostrando como Martí, Quiroga, Andrade e Lispector se expressavam diante dessa temática e quais eram as suas preocupações e posicionamentos para uma revitalização da infância na sociedade. Vale ressaltar, ainda, a forte influência do pensamento médico e higienista dentro das políticas institucionais daquele momento, que centralizava na figura materna os cuidados e as responsabilidades pela saúde da criança, numa clara posição biologizante que vincula a figura da mulher à maternidade.

Retomando o que foi dito no início desta resenha, *A Cruzada das Crianças: Intelectuais, Cultura e Política na América Latina* é um trabalho que nos convida a muitas leituras. Pode ser lido e pesquisado para pensarmos as temáticas ligadas à infância em seu tempo histórico e como o lugar da criança se altera quando se modificam as redes políticas, econômicas e culturais que as atravessam e as constituem.

Por outro lado, o livro nos permite conhecer o trabalho dos escritores pesquisados e o lugar de sua literatura na América Latina, funcionando como um roteiro de suas ideias e como elas foram sendo construídas e modificadas. Por fim, possibilita retomarmos o potencial do livro e da literatura como uma forma de buscarmos diálogos com diversos aspectos e cenários da vida social, apontando para o seu papel relevante e formativo no circuito político, cultural e cotidiano.
